# Post-hospitalisation asthma management in primary care: a retrospective cohort study

**DOI:** 10.3399/BJGP.2023.0214

**Published:** 2024-03-05

**Authors:** Dhanusha Punyadasa, Nikita Simms-Williams, Nicola J Adderley, Rasiah Thayakaran, Adel H Mansur, Krishnarajah Nirantharakumar, Prasad Nagakumar, Shamil Haroon

**Affiliations:** Institute of Applied Health Research, University of Birmingham, Birmingham.; Institute of Applied Health Research, University of Birmingham, Birmingham.; Institute of Applied Health Research, University of Birmingham, Birmingham.; Institute of Applied Health Research, University of Birmingham, Birmingham.; University Hospitals Birmingham; Institute of Inflammation and Ageing, University of Birmingham, Birmingham.; Institute of Applied Health Research, University of Birmingham, Birmingham.; Birmingham Women’s and Children’s Hospital, Birmingham; Institute of Inflammation and Ageing, University of Birmingham, Birmingham.; Institute of Applied Health Research, University of Birmingham, Birmingham.

**Keywords:** asthma, cohort studies, management, post-hospitalisation, primary health care, ethnic and racial minorities

## Abstract

**Background:**

Clinical guidelines recommend that patients admitted to hospital for asthma attacks are reviewed in primary care following hospital discharge.

**Aim:**

To evaluate asthma management in primary care following a hospital admission for asthma and its associations with patient characteristics.

**Design and setting:**

A retrospective cohort study using English primary care data from the Clinical Practice Research Datalink Aurum database and linked Hospital Episode Statistics Admitted Patient Care data.

**Method:**

Patients with asthma aged ≥5 years who had at least one asthma-related hospital admission from 1 January 2017 to 31 December 2019 were included. The primary outcome was a composite of any of the following delivered in primary care within 28 days from hospital discharge: asthma review, asthma management plan, asthma medication prescriptions, demonstration of inhaler technique, or smoking cessation counselling. The association between patient characteristics and delivery of clinical care was assessed using logistic regression.

**Results:**

The study included 17 457 patients. A total of 10 515 (60.2%) patients received the primary outcome within 28 days of hospital discharge. There were 2311 (13.2%) who received an asthma review, 1459 (8.4%) an asthma management plan, 9996 (57.3%) an asthma medication, 1500 (8.6%) a demonstration of inhaler technique, and 52 (1.2% of smokers) had smoking cessation counselling. Patients from Black ethnic minority groups received less of this care (27%–54% lower odds, depending on age). However, short-acting bronchodilator prescriptions in the previous year were associated with an increased likelihood of the primary outcome.

**Conclusion:**

A significant proportion of patients do not receive timely follow-up in primary care following asthma-related admissions to hospital, particularly among Black ethnic minority groups.

## Introduction

Asthma is a common chronic disease in children and adults and is responsible for considerable healthcare use.^1^ In 2020 there were 5.4 million people in the UK living with asthma.^2^ Asthma accounts for 2%–3% of primary care consultations,^3^ 60 000 admissions to hospital with 200 000 bed-days per year, and is estimated to cost £1.1 billion a year to UK health services.^4^ It is also an important cause of poor quality of life.^5^

Asthma-related admissions to hospital remain high in the UK despite efforts to improve care. Clinical guidelines recommend that patients admitted to hospital for asthma attacks are reviewed in primary care within 48 h of hospital discharge^6^^,^^7^ as the risk of subsequent exacerbations is higher among these patients.^8^^,^^9^

In the UK, patients with asthma are mostly followed up in primary care and high-risk patients are expected to receive comprehensive post-exacerbation care including an asthma review, asthma management plan, and assessment of inhaler technique.^7^^,^^10^^,^^11^ Although extensive evaluations of in-hospital asthma care have previously been conducted,^12^ there has been little evaluation of follow-up in primary care after a hospital admission.^13^

The current study aimed to evaluate asthma management in primary care after a hospital admission for asthma and to assess the association with patient characteristics.

## Method

### Study design

A retrospective cohort study was conducted among patients who were admitted to hospital with asthma to assess primary care management following discharge using routinely collected primary and secondary care data. The association between patient characteristics and the likelihood of receiving asthma management in primary care following discharge was also assessed. This was part of the Preventing Unscheduled Hospitalisations in Asthma (PUSH Asthma) study.^14^

### Setting

Primary care data from the Clinical Practice Research Datalink (CPRD) Aurum database were used, which contains longitudinal, routinely collected electronic health record data from UK primary care practices. In total, 16% of the general practices in the UK that use the EMIS clinical information system contribute to this database. CPRD Aurum covers 19% of the UK population and includes information on diagnoses (recorded using SNOMED-CT codes) and drug prescriptions.^15^ The linked Hospital Episode Statistics Admitted Patient Care (HES APC) data were also used, which is coded using International Classification of Diseases 10th revision (ICD-10) codes.^16^ Primary care data extraction was undertaken using the Data Extraction for Epidemiological Research (DExtER) tool.^17^

**Table:** How this fits in

Asthma is a common cause of hospital admissions, and clinical guidelines recommend that patients who have had a hospital admission for asthma are followed up in primary care. Little research has been done on evaluating asthma management in primary care following an asthma-related hospital discharge. This study found that 40% of patients admitted to hospital did not receive asthma management in primary care following hospital discharge, particularly among patients from Black ethnic minority groups. Primary and secondary care services should develop systems for ensuring the timely follow-up of patients with asthma after hospital discharge and address the observed health inequities.

### Participants

Eligible patients were aged ≥5 years with a diagnosis of asthma before 1 January 2017 (index date), who were registered with a general practice contributing to CPRD Aurum at least 1 year before the index date and admitted to hospital for asthma during the study follow-up period (1 January 2017 to 31 December 2019).

Asthma was defined as the presence of a SNOMED-CT code for asthma (see Supplementary Table S1 for SNOMED-CT codes). Asthma-related admission to hospital was defined by the presence of an ICD-10 asthma diagnosis code (J45–J46) as the primary diagnostic code in the linked HES APC data.

Patients who had diagnoses of chronic obstructive pulmonary disease (COPD), bronchiectasis, obstructive sleep apnoea, and interstitial lung disease (ILD) in addition to asthma were excluded from the analysis because of the potential for misclassification bias. Diagnoses were identified as a clinical code for each condition at baseline.

### Baseline variables

Baseline data were extracted to describe demographic characteristics (age, sex, ethnic group, socioeconomic status measured by Index of Multiple Deprivation [IMD] quintiles), behavioural risk factors (body mass index [BMI] and smoking status [for adolescents and adults only]) and relevant comorbidities (allergies, atopic eczema, allergic rhinitis, gastroesophageal reflux disease, chronic rhinosinusitis, anxiety, and depression) before the index date.

Age was determined at the index date. BMI was determined by the latest recorded value before the index date. Where patients had >1 entry for smoking status in the same record the most recent smoking status recorded was used. For rare cases where a patient’s most recent smoking status was ‘non-smoker’ but there was also an ex-smoker/smoker code in the same record, the patient was classed as an ex-smoker.

Asthma-related drug prescriptions within 1 year before the index date were extracted for the following medications: short-acting beta 2 agonists (SABA), oral corticosteroids (OCS), inhaled corticosteroids (ICS), long-acting beta 2 agonists (LABA), long-acting muscarinic antagonists (LAMA), leukotriene receptor antagonists (LTRA), and flu vaccination.

Asthma-related hospital admissions within 1 year before the index date were also extracted.

Clinical code lists for all conditions were created using a systematic process with clinical input that involved checking existing code lists used by the study’s research team, checking published code lists, searching the SNOMED-CT terminology browser, and searching using free-text terms within an in-house software tool called Code Builder. SNOMED-CT code lists for clinical diagnoses are published on GitHub (https://github.com/annalhead/CPRD_multimorbidity_codelists/tree/main/codelists) and in Supplementary Tables S2–S6.

### Outcomes

The following aspects of asthma care were extracted from primary care records for within 48 h, 7 days, and 28 days of hospital discharge: provision of an asthma review, asthma management plan, prescriptions of asthma medications (SABA, OCS, ICS, LABA, LAMA, and LTRA), demonstration of inhaler technique, and smoking cessation counselling. The primary outcome was a record of any of these items of care being recorded within 28 days following the date of hospital discharge.

Changes in inhaler medications post-hospital discharge were also assessed by comparing the drug class of prescriptions recorded in the year before the hospital admission and those prescribed within 28 days after the hospital discharge.

### Study size

The maximum number of eligible patients available in the database was used and study size was not determined by a formal sample size calculation (see Supplementary Figure S1).

### Quantitative variables

Variables were categorised into the following groupings: age (5–11, 12–17, 18–24, 25–39, 40–59, 60–79, and ≥80 years), ethnic group (White, Black, Mixed, Asian, other, and missing), IMD score quintile (1 [least deprived], 2, 3, 4, and 5 [most deprived]), BMI (<18.5 kg/m^2^ [underweight], 18.5–24.9 kg/m^2^ [normal weight], 25–29.9 kg/m^2^ [overweight], ≥30 kg/m^2^ [obesity], and missing), smoking status (current smoker, former smoker, never smoked, and missing), SABA prescriptions (0, 1–3, 4–6, and ≥7 prescriptions), and number of hospital admissions within the previous year (0, 1–3, 4–6, and ≥7).

### Statistical analysis

The cohort were stratified into children (aged 5–11 years), adolescents (aged 12–17 years) and adults (aged ≥18 years). Baseline characteristics and outcomes for each age stratum were described using simple descriptive statistics. Missing data were addressed using a ‘missing’ category for each categorical variable. The primary outcome was presented as the proportion of patients with an asthma-related hospital admission who received asthma management within 28 days of hospital discharge. Secondary outcomes were asthma management within 48 h and 7 days of discharge. The associations between the primary outcome and patient characteristics were assessed using logistic regression, adjusted for demographic and clinical factors. Data analyses were done using Stata SE (version 16) and R Studio.

## Results

### Participants

The study included 17 457 patients who had at least one asthma-related hospital admission during the study period. This included 2512 children, 1511 adolescents, and 13 434 adults. Their baseline characteristics are shown in Table 1.

**Table 1.** Baseline characteristics

**Characteristic**	**Children (*N* = 2512)**	**Adolescents (*N* = 1511)**	**Adults (*N* = 13 434)**
**Age, years, median (IQR)**	8.9 (7.5–10.3)	14.5 (13.2–16.0)	48.9 (33.9–63.9)

**Sex, female, *n* (%)**	893 (35.5)	747 (49.4)	9624 (71.6)

**Ethnicity, *n* (%)**			
White	1323 (52.7)	717 (47.5)	8893 (66.2)
Black	215 (8.6)	105 (6.9)	556 (4.1)
Mixed	136 (5.4)	79 (5.2)	265 (2.0)
Asian	403 (16.0)	200 (13.2)	1375 (10.2)
Other	57 (2.3)	23 (1.5)	167 (1.2)
Missing	378 (15.0)	387 (25.6)	2178 (16.2)

**Index of Multiple Deprivation quintile, *n* (%)**			
1 (least deprived)	369 (14.7)	210 (13.9)	2044 (15.2)
2	355 (14.1)	208 (13.8)	2207 (16.4)
3	400 (15.9)	263 (17.4)	2435 (18.1)
4	555 (22.1)	336 (22.2)	3059 (22.8)
5 (most deprived)	831 (33.1)	494 (32.7)	3681 (27.4)
Missing	2 (0.1)	0 (0)	8 (0.1)

**Body mass index, *n* (%)**			
Underweight	186 (7.4)	203 (13.4)	299 (2.2)
Normal weight	1008 (40.1)	633 (41.9)	3172 (23.6)
Overweight	245 (9.8)	215 (14.2)	3587 (26.7)
Obesity	122 (4.9)	139 (9.2)	5593 (41.6)
Missing	951 (37.9)	321 (21.2)	783 (5.8)

**Smoking status, *n* (%)**			
Current smoker	—	176 (11.6)	4093 (30.5)
Former smoker	—	193 (12.8)	5296 (39.4)
Never smoked	—	921 (61.0)	3818 (28.4)
Missing	—	221 (14.6)	227 (1.7)

**Comorbidities, *n* (%)**			
Allergies	714 (28.4)	605 (40.0)	4505 (33.5)
Atopic eczema	1392 (55.4)	871 (57.6)	4205 (31.3)
Allergic rhinitis	529 (21.1)	569 (37.7)	4344 (32.3)
Gastro-oesophageal reflux disease	162 (6.4)	76 (5.0)	2328 (17.3)
Chronic rhinosinusitis	0 (0)	3 (0.2)	425 (3.2)
Anxiety	20 (0.8)	104 (6.9)	4407 (32.8)
Depression	22 (0.9)	59 (3.9)	6432 (47.9)

**Medication use within previous year, *n* (%)**			
Short-acting beta 2 agonist			
0 prescriptions	323 (12.9)	227 (15.0)	3022 (22.5)
1–3 prescriptions	1055 (42.0)	488 (32.3)	4213 (31.4)
4–6 prescriptions	604 (24.0)	316 (20.9)	2530 (18.8)
≥7 prescriptions	530 (21.1)	480 (31.8)	3669 (27.3)
Oral corticosteroid	990 (39.4)	565 (37.4)	7018 (52.2)
Inhaled corticosteroid	1973 (78.5)	1193 (79.0)	10 382 (77.3)
Long-acting beta 2 agonist	55 (2.2)	78 (5.2)	2760 (20.5)
Long-acting muscarinic antagonist	0 (0)	7 (0.5)	1206 (9.0)
Leukotriene receptor antagonist	804 (32.0)	482 (31.9)	2608 (19.4)
Flu vaccine	1025 (40.8)	564 (37.3)	6920 (51.5)

**Hospital admissions, *n* (%)**			
0	2254 (89.7)	1385 (91.7)	12 865 (95.8)
1–3	237 (9.4)	98 (6.5)	469 (3.5)
4–6	14 (0.6)	7 (0.5)	41 (0.3)
≥7	7 (0.3)	21 (1.4)	59 (0.4)

*IQR = interquartile range.*

The median age was 8.9 years (interquartile range [IQR] 7.5–10.3) for children, 14.5 years (IQR 13.2–16.0) for adolescents, and 48.9 years (IQR 33.9–63.9) for adults. Of all 17 457 patients, 11 264 (64.5%) were female. The majority (62.6%) were of White ethnicity followed by 1978 (11.3%) Asian and 876 (5.0%) Black ethnic groups; 2943 (16.9%) had missing ethnicity data. In total, 5006 (28.7%) were from the most deprived socioeconomic quintile (Table 1).

Of the 17 457 patients, there were 9901 (56.7%) with overweight or obesity. There were 176 (11.6%) adolescents and 4093 (30.5%) adults who were current smokers. The most common comorbidity was atopic eczema for children (*n* = 1392/2512 [55.4%]) and adolescents (*n* = 871/1511 [57.6%]), and depression for adults (*n* = 6432/13 434 [47.9%]) (Table 1).

Within the previous year (2016), of the 17 457 patients there were 13 885 (79.5%) who had received a SABA, 13 548 (77.6%) an ICS, 8573 (49.1%) an OCS, and 3894 (22.3%) an LTRA. In total, 8948 (51.3%) had not received a flu vaccine within the previous year. There were 953 (5.5%) patients who had experienced ≥1 asthma-related hospital admissions within the previous year (Table 1).

### Outcomes

**Figure 1.**
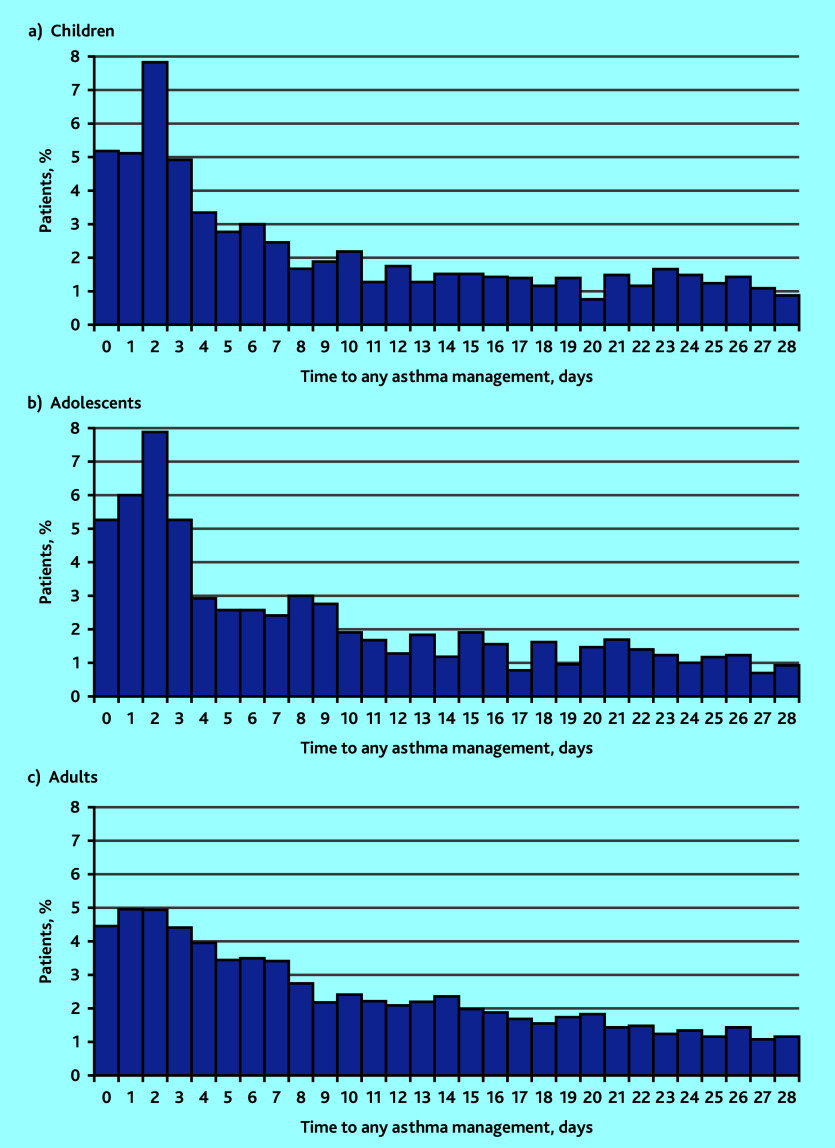
Percentage of patients receiving any asthma management in primary care after a asthma-related hospital admission from the time of hospital discharge in a) children, b) adolescents and c) adults.

Asthma management following discharge is summarised in Table 2 and Figure 1. Out of 17 457 patients, 10 515 (60.2%) had the primary outcome within 28 days of hospital discharge. In total, 2311 (13.2%) had received an asthma review, 1459 (8.4%) an asthma management plan, 9996 (57.3%) an asthma medication, 1500 (8.6%) a demonstration of inhaler technique, and 52 (1.2% of smokers) had smoking cessation counselling.

**Table 2.** Asthma management received in primary care after a discharge for an asthma-related hospital admission^a^

**Asthma management**	**Children (*N* = 2512)**			**Adolescents (*N* = 1511)**			**Adults (*N* = 13 434)**		
	**Timeframe within:**			**Timeframe within:**			**Timeframe within:**		
	**48 h**	**7 days**	**28 days**	**48 h**	**7 days**	**28 days**	**48 h**	**7 days**	**28 days**
**Any asthma management^b^**	453 (18.0)	834 (33.2)	1473 (58.6)	270 (17.9)	483 (32.0)	880 (58.2)	1862 (13.9)	4262 (31.7)	8162 (60.8)
**Asthma management plan^b^**	34 (1.4)	94 (3.7)	257 (10.2)	20 (1.3)	57 (3.8)	156 (10.3)	122 (0.9)	375 (2.8)	1046 (7.8)
**Asthma review^b^**	59 (2.3)	145 (5.8)	391 (15.6)	31 (2.1)	85 (5.6)	222 (14.7)	199 (1.5)	590 (4.4)	1698 (12.6)
**Inhaler technique^b^**	20 (0.8)	66 (2.6)	227 (9.0)	11 (0.7)	43 (2.8)	138 (9.1)	99 (0.7)	343 (2.6)	1135 (8.4)
**Smoking cessation^c^**	N/A	N/A	N/A	0 (0)	3 (1.7)	3 (1.7)	15 (0.4)	29 (0.7)	49 (1.2)
**Any medication^b^**	426 (17.0)	779 (31.0)	1391 (55.4)	251 (16.6)	447 (29.6)	828 (54.8)	1713 (12.8)	3943 (29.4)	7777 (57.9)
**Short-acting beta 2 agonist**	298 (11.9)	570 (22.7)	1073 (42.7)	180 (11.9)	327 (21.6)	670 (44.3)	212 (1.6)	2137 (15.9)	5127 (38.2)
**Inhaled corticosteroid**	219 (8.7)	423 (16.8)	927 (36.9)	147 (9.7)	276 (18.3)	603 (39.9)	925 (6.9)	2251 (16.8)	5848 (43.5)
**Oral corticosteroid**	110 (4.4)	171 (6.8)	267 (10.6)	65 (4.3)	106 (7.0)	169 (11.2)	551 (4.1)	1408 (10.5)	2551 (19.0)
**Long-acting muscarinic antagonist**	0 (0)	0 (0)	0 (0)	0 (0)	1 (0.1)	5 (0.3)	85 (0.6)	247 (1.8)	799 (5.9)
**Long-acting beta 2 agonist**	5 (0.2)	9 (0.4)	18 (0.7)	9 (0.6)	16 (1.1)	43 (2.8)	253 (1.9)	676 (5.0)	1908 (14.2)
**Leukotriene receptor antagonist**	83 (3.3)	172 (6.8)	435 (17.3)	32 (2.1)	69 (4.6)	220 (14.6)	209 (1.6)	578 (4.3)	1656 (12.3)

a *Data are* n *(%).*

b *Denominator is total number of patients in each age classification (children, adolescents, and adults).*

c *Denominator is number of patients who are current smokers in that age classification (total* n *= 176 for adolescents and total* n *= 4093 for adults). N/A = not applicable (as smoking status was unavailable for children).*

Of the 2512 children, 1473 (58.6%) received at least one form of asthma management within 28 days of hospital discharge. This figure was lower within 7 days (*n* = 834/2512 [33.2%]) and 48 h (*n* = 453/2512 [18.0%]) after hospital discharge. A total of 257 (10.2%) received an asthma management plan, 391 (15.6%) had an asthma review, and 227 (9.0%) had a demonstration of inhaler technique within 28 days of hospital discharge. There were 1391 (55.4%) children who received at least one asthma medication prescription within 28 days of hospital discharge, of which SABA was the most prescribed (*n* = 1073/2512 [42.7%]) (Table 2).

Of the 1511 adolescents, 880 (58.2%) received some form of asthma management within 28 days of hospital discharge. Again, this was lower within 7 days (*n* = 483/1511 [32.0%]) and 48 h (*n* = 270/1511 [17.9%]) from hospital discharge. A total of 156 (10.3%) had been provided with an asthma management plan, 222 (14.7%) had an asthma review, 138 (9.1%) had a demonstration of inhaler technique, and only three (1.7%) patients who currently smoked were offered smoking cessation counselling within 28 days of hospital discharge. There were 828 (54.8%) adolescents who received asthma medication prescriptions within 28 days of hospital discharge, with SABA again being the most prescribed (Table 2).

Of the 13 434 adults, 8162 (60.8%) received some form of asthma management within 28 days of hospital discharge. Again, this was lower within 7 days (*n* = 4262/13 434 [31.7%]) and 48 h (*n* = 1862/13 434 [14.0%]) from hospital discharge. A total of 1046 (7.8%) had been provided with an asthma management plan, 1698 (12.6%) had an asthma review, 1135 (8.4%) had a demonstration of inhaler technique, and only 49 (1.2%) of those who currently smoked had been offered smoking cessation counselling within 28 days of hospital discharge. There were 7777 (57.9%) adults who received asthma medication prescriptions within 28 days of hospital discharge, with ICS being the most prescribed (Table 2).

A total of 8.8% of children (*n* = 220/2512) and 8.9% of adolescents (*n* = 134/1511) had a change in their inhaler medication within 28 days of hospital discharge (see Supplementary Table S7), with the main change being the addition of ICS. For adults, 13.7% (*n* = 1840/13 434) had a change in their inhaler medication within 28 days of hospital discharge, with the main change being the addition of a LABA.

### Factors associated with asthma management within 28 days of hospital discharge

In all age cohorts, Black ethnic minority groups were the least likely to receive any asthma management within 28 days of hospital discharge (odds ratio [OR] 0.62, 95% confidence interval [CI] = 0.45 to 0.86 for children; OR 0.46, 95% CI = 0.29 to 0.73 for adolescents; and OR 0.73, 95% CI = 0.60 to 0.89 for adults) (see Supplementary Table S8).

Adolescents with obesity were less likely than those of normal weight to receive asthma management within 28 days of hospital discharge (OR 0.60, 95% CI = 0.40 to 0.90). By contrast, adults with obesity had greater odds of receiving asthma management within 28 days of hospital discharge than patients of normal weight (OR 1.13, 95% CI = 1.02 to 1.25) (see Supplementary Table S8).

Among adults, patients aged ≥25 years had an increased odds of receiving asthma management within 28 days of hospital discharge, compared with those aged 18–24 years. This was most pronounced for patients aged 60–79 years who had an 82% increase in the odds of receiving asthma care 28 days after hospital discharge (OR 1.82, 95% CI = 1.56 to 2.12) (see Supplementary Table S8).

Children who had received SABA and LTRA prescriptions in the previous year were more likely to receive asthma management within 28 days of hospital discharge than those who had not received prescriptions in the previous year. Those who had received 1–3 SABA prescriptions in the previous year had almost a threefold increase in the odds of receiving asthma care following hospital discharge (OR 2.77, 95% CI = 2.00 to 3.84) whereas those who had received ≥7 prescriptions in the previous year had a more than sixfold increase in the odds (OR 6.44, 95% CI = 4.29 to 9.64). A similar dose–response trend for SABAs was also found for adolescents and adults. In adults, positive associations were also found for previous prescriptions of OCS, ICS, LTRA, and flu vaccine (see Supplementary Table S8).

## Discussion

### Summary

The current analysis of 17 457 patients with asthma revealed that a substantial proportion did not receive timely asthma management in primary care following an asthma-related discharge from hospital. The majority of patients, 85%, had not received primary care-based asthma management within 48 h of hospital discharge, and 40% had not within 28 days, including an asthma review, asthma management plan, relevant prescriptions, demonstration of inhaler technique, or smoking cessation support. Patients from Black ethnic minority groups were the least likely to receive any asthma care following their discharge. Care was also less likely to be received by adolescents with obesity and younger adults. Patients who had received asthma medication prescriptions in the previous year were more likely to receive care following discharge in primary care.

### Strengths and limitations

The large sample size allowed the authors to stratify the analysis by three age classifications — children, adolescents, and adults. This enabled the identification of disparities in asthma care post-hospital discharge in each age classification and the risk factors associated with differences in primary care management after hospital discharge.

Asthma was defined by the presence of prespecified SNOMED-CT codes in primary care records. Asthma is a clinical diagnosis that can often be misdiagnosed in primary care.^18^^,^^19^ Asthma-related admissions to hospital could also be misclassified as there may be overlapping symptoms of exacerbations because of other comorbidities. This misclassification bias was minimised by excluding patients with asthma who also had other chronic respiratory diseases such as COPD, bronchiectasis, and ILD.

A significant proportion of hospital attendances with asthma attacks are made to the emergency department, but these data were not available for use in the current study except for when patients were admitted. The assessment of clinical management was also limited to using clinical codes corresponding to asthma management, which would not incorporate aspects of care that were documented through free-text entries in primary care records. This may have resulted in an underestimate of asthma care after discharge; however, the authors expected most asthma care to be well coded as asthma is part of the Quality and Outcomes Framework in England, a payment incentive programme rewarding appropriate reporting and management of specific chronic conditions. Any underestimate is therefore likely to be small.

A further limitation was a lack of data on asthma care delivered before hospital discharge. This information may be included in discharge letters to primary care and influence the necessity of rapid follow-up, which it was not possible to adjust for. Future work could address this limitation by linking data from the national asthma audit, which collects data on hospital care.

It was possible to explore a range of patient characteristics in the current analysis. However, it is likely that there are additional factors that may be associated with asthma care following an asthma-related admission to hospital that were not explored in the current study, for instance, practice-level characteristics including rural/urban location and practice size, and additional patient-level characteristics such as multimorbidity and frailty. Despite adjusting for several potential confounders, because of the cohort study design, it is possible that some results may be affected by residual confounding.

The current study’s follow-up ended in 2019. Future studies should explore the impact of changes in healthcare delivery during and after the COVID-19 pandemic on asthma reviews after an asthma-related admission to hospital. The impact of telephone-based asthma reviews in particular needs to be evaluated as essential asthma care assessment, including checking inhaler technique and whether inhalers are empty,^20^ cannot be adequately performed via a telephone review.

### Comparison with existing literature

Multiple studies have evaluated asthma management in UK primary care.^21^^–^^23^ To the authors’ knowledge, this study is the first to evaluate asthma management in a large UK primary care population following asthma-related hospital admission. Although evidence suggests comprehensive asthma care following discharge from hospital minimises adverse asthma outcomes,^24^^,^^25^ the current study found that the practice of such care was significantly below existing standards and guidelines.^7^^,^^10^ Indeed, 85% of patients with asthma failed to receive follow-up asthma care within the recommended 48 h of hospital discharge^7^^,^^10^ in the current study, which is consistent with previous evidence.^26^^,^^27^ The UK National Review of Asthma Deaths reported that only 23% of patients with asthma who had died had ever received an asthma management plan from primary or secondary care.^24^ In a survey of Scottish patients who had asthma attacks requiring OCS or admission to hospital within the previous 6 months, only 4% reported receiving a written asthma action plan from primary care.^28^

Ethnic disparities in the provision of asthma care have been reported by several previous studies^22^^,^^29^ and this is also a significant finding in the current study, particularly among Black ethnic groups. Previous literature suggests that this disparity is because of differences in health-seeking behaviour^30^ and less familiarity with primary healthcare services^31^ among these population groups. Given that these population groups exhibit much higher asthma-related admissions to hospital and emergency department visits,^29^^,^^32^ these disparities need urgent intervention. Hospital teams should address essential asthma care and make changes to medications before discharging patients. GPs, practice nurses, and school nurses should use every opportunity to educate, review, and change asthma management to prevent future asthma attacks.

Patients with asthma who are in socioeconomically deprived cohorts tend to have relatively poor asthma outcomes;^9^^,^^22^^,^^33^^,^^34^ however, receiving asthma care after discharge for an asthma-related hospital admission was not significantly associated with socioeconomic deprivation in the current study. A previous UK study also reported no variation in receiving asthma reviews or asthma referrals by socioeconomic status of patients.^22^ By contrast, Alsallakh *et al* reported that patients with asthma in Wales who were the most socioeconomically deprived had lower levels of asthma-related primary care consultations and prescribing, although they did not specifically describe post-hospital admission asthma management.^35^ Further research is needed to assess asthma management in primary care following discharge across socioeconomic strata in other populations to assess the consistency of the current findings.

Despite evidence indicating that obesity is associated with an increased risk of asthma-related admission and readmission to hospital,^36^^–^^38^ the current study found that adolescents with obesity were less likely to receive asthma care after discharge compared with adolescents without obesity. This runs counter to previous literature that suggests that both elective and emergency healthcare utilisation is higher in children with obesity including those with asthma.^39^ It is unclear whether the current findings were the result of chance as the sample size of adolescents with obesity in this study was relatively small (*n* = 139), and future studies should assess whether this is a consistent finding in other populations.

A study from the US evaluating outpatient follow-up care after an adult emergency department asthma visit reported that patients aged ≥45 years were more likely to receive follow-up care within 30 days of an emergency department visit.^26^ A similar increased likelihood for older adults to receive asthma care following discharge compared with younger adults was observed in the current study.

### Implications for research and practice

The current study provides valuable insights into the state of primary care-based asthma management in England following an asthma-related hospital discharge. The study identified significant shortfalls in implementing recommendations for primary care from the national asthma guideline^40^ and National Review of Asthma Deaths,^41^ particularly for Black ethnic minority groups. Improved communication, data sharing, and integrated clinical pathways are needed between secondary and primary care services so that patients with asthma who are admitted to hospital are followed up appropriately in primary care and receive timely interventions.

GPs and practice nurses should receive a clinical handover from secondary care within 24 h of a patient with asthma being discharged from hospital. This information should ideally be structured, containing reconciled discharge information,^42^ and be aligned with national standards for discharge summaries.^43^ Critically, this should include a summary of the hospital admission, including disease severity and treatments given; relevant findings from clinical investigations; reconciled medications and changes to asthma treatment; patient and carer concerns, expectations, and wishes; information and advice given; an updated asthma management plan; and specific actions for the primary care team.^43^ This would facilitate the timely review of these patients in the community to help prevent further exacerbations and improve continuity of care.^44^ This will be particularly important in high-risk patients, such as those from Black ethnic minority groups, who experience significant health inequalities. Patients and their caregivers should also be empowered to actively contact primary care services when they are discharged from hospital to ensure they receive timely care. The impact of these service changes could be evaluated by repeating this study in future cohorts and incorporating data on emergency department attendance.

In conclusion, there are significant shortfalls and inequalities in asthma care in general practice in England following an asthma-related hospital discharge. Robust systems are needed to ensure the timely follow-up of patients with asthma in primary care following asthma-related hospital discharge, which includes timely handover from secondary care.

## Data

Access to anonymised patient data from CPRD is subject to a data sharing agreement containing detailed terms and conditions of use following protocol approval from the Medicines and Healthcare products Regulatory Agency Independent Scientific Advisory Committee. This study-specific analysable dataset is therefore not publicly available, but can be requested from the corresponding author, subject to research data governance approvals. Details about Independent Scientific Advisory Committee applications and data costs are available on the CPRD website (https://cprd.com).
